# Transmission-ratio distortion in the Framingham Heart Study

**DOI:** 10.1186/1753-6561-3-s7-s51

**Published:** 2009-12-15

**Authors:** Andrew D Paterson, Daryl Waggott, Arne Schillert, Claire Infante-Rivard, Shelley B Bull, Yun Joo Yoo, Dushanthi Pinnaduwage

**Affiliations:** 1 Program in Genetics and Genome Biology, Hospital for Sick Children, 101 College Street, TMDT East Tower, Toronto, ON M5G 1X8 Canada; 2 Dalla Lana School of Public Health, University of Toronto, 155 College Street, Toronto, Ontario M5T 3M7 Canada; 3 Samuel Lunenfeld Research Institute of Mount Sinai Hospital, Prosserman Centre for Health Research, 60 Murray Street, Toronto, ON M5T 3L9 Canada; 4 Institut für Medizinische Biometrie und Statistik, Universität zu Lübeck, Maria-Goeppert Str. 1, 23562 Lübeck, Germany; 5 Department of Epidemiology, Biostatistics and Occupational Health, Faculty of Medicine, McGill University, 1110 Pine Avenue West, Montréal, Québec H3A 1A3 Canada

## Abstract

Transmission-ratio distortion (TRD) is a phenomenon in which the segregation of alleles does not obey Mendel's laws. As a simple example, a recessive locus that results in fetal lethality will result in live-born individuals sharing more alleles at this locus than expected under Mendel's laws. This could result in apparent linkage of the phenotype of 'being alive' to such a chromosomal regions. Further, this could result in false-positive linkage when 'affected-only' parametric or non-parametric linkage analysis is performed. Similarly, loci demonstrating TRD may be detectable in family-based association tests as deviant transmission of alleles. Therefore, TRD could result in confounding of family-based association studies of diseases. The Framingham Heart Study data available for Genetic Analysis Workshop 16 is a suitable dataset to determine whether there are loci in the genome that reveal TRD because of the large number of individuals from families, the high-resolution genotyping, and the population-based nature of the study. We have used both genome-wide linkage and family-based association methods to determine whether there are loci that demonstrate TRD in the Framingham Heart Study. Family-based association analysis identified thousands of loci with apparent TRD. However, the vast majority of these are likely the result of genotyping errors with application of strict quality control criteria to the genotype data, and automated inspection of the intensity plots, we identify a small number of loci that may show true TRD, including rs1000548 in intron 6 of S-antigen (arrestin, SAG) on chromosome 2 (*p *= 7 × 10^-10^).

## Background

A critical assumption for the majority of genetic mapping approaches (including both linkage and family-based association) is that Mendel's law of segregation is obeyed. Transmission-ratio distortion (TRD) refers to the deviation from the expected Mendelian inheritance of alleles. Violation of this assumption could result in false-positive linkage, particularly within 'affected-only' or 'non-parametric' linkage analysis frameworks. Futhermore, within a family-based association design, the presence of TRD could produce spurious association if transmissions are only assessed to affected, but not unaffected offspring. In addition, it is feasible that the presence of TRD could also reduce the power to detect true disease loci. The presence of TRD in humans has been addressed in only a few studies, using either linkage [1] or family-based association methods [2]. However, these studies had limited sample sizes, which may have resulted in low power. This limitation has recently been emphasized, when it was shown that hundreds or thousands of trios would be needed to detect loci even with large TRD deviations [3].

For a variety of reasons, including that of statistical power, the majority of genome-wide association studies have used a case-control design, which is not able to detect loci that are subject to TRD. However, some studies are employing a family-based design, but it is typical for them to study only affected offspring, and they are thus susceptible to identifying loci that demonstrate TRD and falsely concluding that they are associated with the disease of interest. Unless unaffected sibs are genotyped, one cannot determine whether association signals are the result of confounding by TRD. Therefore, we took advantage of the large sample size, pedigree-based design and genome-wide genotyping of the Framingham Heart Study Problem 2 data from Genetic Analysis Workshop 16 (GAW16) to determine whether we could identify loci demonstrating TRD.

## Methods

### Subject and genotype data

We used data from Affymetrix 500 k and 50 k single-nucleotide polymorphism (SNP) datasets from Problem 2 of GAW16, the Framingham Heart Study. Genotype data were called by the data providers using BRLMM [4], but no details were provided about how samples were batched for genotype calling. Data providers removed relationship errors and sample mix-ups but not any remaining Mendelian errors.

### Linkage analysis

All genotyped individuals in the last generation were coded as 'affected' and we used non-parametric linkage approaches (Cox and Kong non-parametric linkage (NPL)) to determine whether there are regions in the genome linked to the phenotype of 'being alive in the last generation' (Merlin v 1.1.2) [5]. We dealt with linkage disequilibrium among the ~500 k SNPs by selecting a subset of SNPs based on: minor allele frequency (MAF)>45%, Hardy-Weinberg equilibrium (HWE) *p*-value > 0.05, individual genotype missing rate <5%, SNP missing rate <2%, pairwise *r*^2 ^< 0.05, and Mendelian error rate <5%. Individuals from Cohort 1 were not used in the analysis, therefore large pedigrees were split into smaller pedigrees using the R kinship package (makefamid function [6]) to allow the computation of NPL statistics.

### Family-based association analysis

We also performed family-based association tests (i.e., the transmission-disequilibrium test, or TDT) to examine the transmissions of alleles for all SNPs across the genome to all genotyped individuals in the dataset using PLINK v1.02 [7,8] with the Affymetrix 500 k and HuGeneFocused 50 k SNP genotype data. SNPs were initially selected to have MAF>1%, call rates >90%, and HWE *p *> 10^-5^.

## Results

### Linkage analysis

Genome-wide linkage analyses used ~5 k SNPs from 1,028 pedigrees that were informative. There were no loci that met genome-wide criteria for significant linkage.

### Family-based association analysis

Genome-wide TDT analysis was performed and identified 2,722 autosomal SNPs with TDT *p *< 10^-8^, which was an unexpectedly large number. However, when we investigated this further, we suspected that the majority of these results were false positives due to genotyping error. It has been reported previously that, in the presence of certain common types of genotyping error, there is a bias to excess transmission of the major as opposed to minor allele for SNPs [9]. Indeed, in this data there was a striking bias in the transmission rates based on whether the major or minor allele showed excess transmission. Specifically, there were 2,701 SNPs with TDT *p *< 10^-8^, HWE *p *> 10^-5^, and MAF>1% in which the major allele showed excess transmission. This compared to only 21 SNPs using the same criteria in which the minor allele showed excess transmission.

To confirm our suspicions that genotyping error was the major cause of the large number of positive results, we took advantage of the fact that it is more difficult to detect Mendelian errors for SNPs with lower MAF [10]. This would lead us to expect that low-allele-frequency SNPs would be disproportionately represented in those SNPs that demonstrate excess transmission of the major allele compared to those where the minor allele showed excess transmission. Consistent with this expectation, when we compared the MAF as a function of the transmission of the major or minor allele for these 2,722 SNPs, the MAF was significantly lower for those SNPs where the major allele showed over-transmission (3.8 ± 4.4%, mean ± SD) compared with those where the minor allele was over-transmitted (33% ± 12%, *p *< 0.0001).

**Figure 1:**
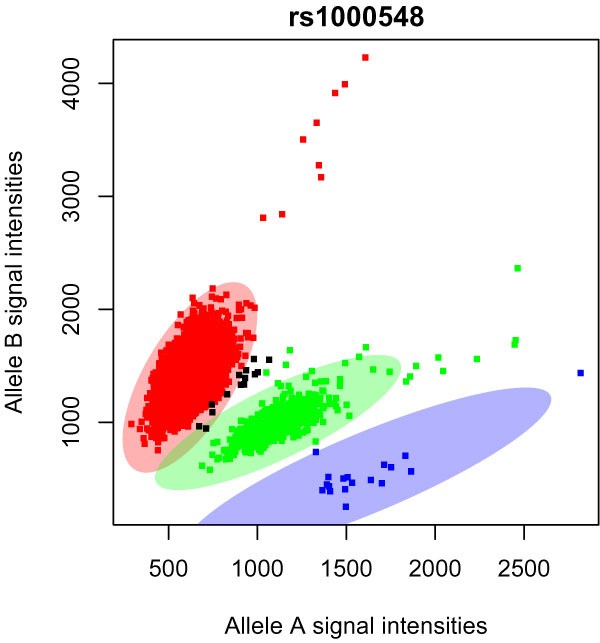
**Clusterplot for rs1000548**. The X and Y axis are the intensities of the two alleles at this SNP. The red, green, and blue squares are the intensities for individuals who were called common homozygous, heterozygous, and rare homozygous genotypes, respectively. The black squares represent individuals who have missing genotypes. The colored ellipses, defined by ACPA, are the regions in which only samples of that genotype are expected.

Visual inspection of the cluster-plots of thousands of SNPs is labor intensive, so we next investigated whether we could use automated methods to help distinguish which SNPs had good quality genotype calls. We then applied a less stringent criteria for TRD (i.e., *p *< 10^-5^), and for these 4,501 SNPs we ran automated cluster plot analysis (ACPA) [11]. We limited this analysis to SNPs with MAF >0.01, missing rate < 0.02, and HWE *p*-value>10^-4^. Using a criteria for genome-wide significance of *p *< 10^-8^, only one SNP was predicted by the ACPA procedure to have good quality genotype clustering, rs1000548 (TDT *p *= 7.4 × 10^-10^; Figure 1). Details about this and other SNPs that were also predicted using ACPA to have good quality genotype clustering using a more relaxed significance criteria (TDT *p *< 10^-5^) are provided in Table 1. For these 8 SNPs, there was no significant heterogeneity between the paternal and maternal transmission rates (*p *> 0.08).

**Table 1:** SNPs showing TRD (TDT *p *< 10^-5^) with genotype clustering passing ACPA

SNP	Chr^a^	Nucleotide position in build 36	T^b^	U^c^	OR^d^	TDT *p*-value	Minor:major allele	MAF^e^	HWE *p*^f^	MISS^g^	Mendel error count	Nearest gene(s) and location
rs12091564	1	144,106,961	103	178	0.58	7.7 × 10^-6^	C:T	0.06	0.70	0.010	0	Intergenic *NBPF8 *and *HFE2*
rs17015803	2	119,907,901	58	124	0.47	1.0 × 10^-6^	C:T	0.04	0.13	0.014	1	*TMEM37 *intron 1
rs1000548	2	233,900,041	107	218	0.49	7.4 × 10^-10^	C:T	0.07	0.09	0.011	3	*SAG *intron 6
rs17684740	5	126,748,314	28	79	0.35	8.2 × 10^-7^	C:T	0.04	0.66	0.009	1	*MEGF10 *intron 6
rs757128	5	136,648,485	27	76	0.36	1.4 × 10^-6^	A:G	0.03	0.42	0.016	0	*SPOCK1 *intron 2
rs7029570	9	96,722,609	59	130	0.45	2.4 × 10^-7^	G:A	0.05	0.17	0.015	1	*C9orf3 *intron 5
rs1156214	9	121,995,064	535	394	1.36	3.7 × 10^-6^	G:T	0.25	0.02	0.002	4	*DBC1*, *CDK5RAP2*, *MEGF9*
rs3786228	18	75,565,158	500	663	0.75	1.8 × 10^-6^	C:T	0.37	0.09	0.002	0	*CTDP1 *intron 4

^a^Chr, chromosome

^b^T, counts of transmission of the minor allele

^c^U, counts of non-transmissions of the minor allele

^d^OR, odds ratio for transmission of the major allele

^e^MAF, minor allele frequency

^f^HWE *p*, Hardy Weinberg equilibrium *p*-value

^g^MISS, frequency of missing genotype data

## Discussion and conclusion

The results of TDT analyses performed here have highlighted the problems of using high-throughput genotype data with even a small proportion of genotyping errors to detect phenomenon such as TRD. The gross over-transmission of the common allele for SNPs with a pattern consistent with TRD, and the marked allele frequency difference between them and the SNPs where the minor allele shows excess transmission, are consistent with genotyping error being the major force behind the unexpectedly large number of apparent positive results. Further contributing to the bias described by Mitchell et al. [9] in which genotyping errors are more difficult to detect for SNPs with low MAF, is the concern that the genotype error rate for rarer SNPs may be higher due to batch-calling of genotypes. These concerns make it challenging to distinguish true effects from artifact. Alternative genotype calling algorithms, which call genotypes from all or larger sets of samples at once, or even across multiple studies, have been shown to improve the quality of genotype calling, e.g., CHIAMO [12]. In addition, this work has implication for implementation of imputation strategies for ungenotyped SNPs (which is common for genome-wide association studies). Because we found that >1% of SNPs in this dataset likely have poor quality genotype calling even after applying conventional quality control criteria, this means that ungenotyped SNPs that are imputed based on these SNPs which have genotyping errors are likely to be subject to considerable error.

In addition to the complexities that have arisen in the interpretation of our analysis, there is concern that the use of HWE as a criterion to filter SNPs for the analysis of TRD is a double-edged sword. Some SNPs showing true TRD may also deviate significantly from HWE because of violation of the selection assumption, and may end up being removed from datasets in an attempt to remove genotyping errors. Similarly, automatic exclusion of SNPs with low MAF may bias against the detection of true TRD loci because it is likely that because of negative selection, SNPs which show TRD tend to have low MAF. Another caveat of this study is that at each of the eight regions with evidence for TRD (Table 1), there is only one SNP in each region which shows evidence for TRD. Given the general selection of SNPs on the Affymetrix 500 k chip, we would expect that in some regions there would be other SNPs with similar TRD results, so this makes us cautious about over-interpretation of these results.

There are some interesting genes near the SNPs in Table 1 that show TRD. For example, rs3786228 is in intron 4 of *CTDP1 *(carboxy-terminal domain, RNA polymerase II, polypeptide A phosphatase, subunit 1) on chromosome 18; autosomal recessive mutations in this gene have been shown to results in 'congenital cataracts facial dysmorphism neuropathy' (*CCFDN*), a developmental disorder prevalent in Roma Gypsies [13]. Similarly, autosomal recessive inheritance of mutations in *SAG *(S-antigen, arrestin) have been found in Oguchi disease, a rare autosomal recessive form of night blindness [14]. In this study we observed marked TRD of rs1000548, in intron 6 of *SAG*. It may be that in populations similar to Framingham, variation in these genes contributes to phenotypes that can result in TRD, including the failure of fertilization, implantation, or the differential survival of fetuses. Identifying loci that demonstration TRD could provide insight into the mechanisms of the processes.

## List of abbreviations used

ACPA: Automated cluster plot analysis; GAW: Genetic Analysis Workshop; HWE: Hardy-Weinberg equilibrium; MAF: Minor allele frequency; NPL: Non-parametric linkage; TDT: Transmission-disequilibrium test; TRD: Transmission-ratio distortion; SNP: Single-nucleotide polymorphism;

## Competing interests

The authors declare that they have no competing interests.

## Authors' contributions

ADP, CI-R and SBB conceived of the idea. DW, DP, and YJY performed the linkage and association analysis. AS ran the ACPA analysis. ADP wrote a draft of the manuscript which all authors edited, read and approved the final manuscript.
